# Clinical importance of incisional hernia in patients resected for colorectal liver metastases: quality of life and abdominal wall symptoms

**DOI:** 10.1007/s00423-025-03638-3

**Published:** 2025-02-12

**Authors:** Peter Strandberg Holka, Gert Lindell, Bobby Tingstedt, Christian Sturesson

**Affiliations:** 1 Department of Clinical Sciences Lund, Surgery, Lund University, Skane University Hospital, Lund, Sweden; 2 Division of Surgery and Oncology, Department of Clinical Science, Intervention and Technology (CLINTEC), Department of Surgery, Karolinska Institutet and Karolinska University Hospital, S-141 86 Stockholm, Stockholm, Sweden

**Keywords:** Incisional hernia, Liver resection, Colorectal metastases

## Abstract

**Purpose:**

Incisional hernia (IH) after open liver surgery is a well-recognized complication. The clinical importance of IH detected on computed tomography in terms of objective abdominal wall discomfort and impairment of quality of life (QoL) is less well known.

**Methods:**

Patients who underwent curative surgery for colorectal liver metastases between 2010 and 2015 at a single center and were alive in February 2017 were asked to complete a ventral hernia pain questionnaire and the EORTC QLQ-C30 QoL questionnaire.

**Results:**

A total of 105 patients (80%) completed the questionnaires. Forty-three patients (42%) developed IH. The majority (77%) of IHs were < 2.5 cm. Patients who had an IH before liver surgery developed a new IH to a greater extent (*P* = 0.001). There were no significant differences regarding abdominal wall symptoms and QoL between patients with and without IH. However, about half (48%) of all patients had abdominal wall symptoms after a median follow-up of 34 months.

**Conclusion:**

Radiologically detected IH after open liver surgery has low clinical importance. About half of all patients who underwent liver surgery experienced abdominal wall symptoms a long after surgery, but these symptoms were not related to IH.

## Introduction

Incisional hernia (IH) after laparotomy is a common complication. Factors reported to be associated with an increased incidence of IH after resection of colorectal liver metastases include gender, age, obesity, and prolonged preoperative chemotherapy [1, 2, 3, 4]. The reported incidence of IH varies depending on the type of incision, follow-up time, and method of hernia detection [1, 3, 5]. IH has been shown to be less frequent after laparoscopic operations compared to open surgery [6]. Although laparoscopic liver resections are feasible and are increasingly used, open resection is still the most common procedure [7]. Imaging diagnostics increases the ability to detect IH compared to physical examination [5] and computed tomography (CT) has been suggested as “golden standard”. However, the clinical importance of hernias found on CT is unknown. Studies of quality of life (QoL) for patients operated on for colorectal liver metastases show deterioration of QoL after surgery, but patients usually return to baseline within 3 months [8, 9].

With improved survival over time [10], the focus on long-time perspectives, including rehabilitation and QoL, is increasing. In this context, symptoms related to the surgical procedure itself are essential to investigate. Therefore, this study aimed to analyze abdominal wall discomfort and QoL with reference to IH after open liver surgery for colorectal liver metastases.

## Materials and methods

Patients from Skåne, in the southern region in Sweden, who underwent curative surgery for colorectal liver metastases between January 2010 and December 2015 at the department of surgery at Skåne University Hospital, Lund, were screened for inclusion. Patients alive on February 1, 2017 were sent the Swedish version of the EORTC QLQ-C30 [11] and an abdominal wall specific questionnaire [12] by regular mail, along with an informed consent form, and were asked to participate in this study. Patients who did not respond were reminded twice. Those who returned both questionnaires formed the study population.

Data were obtained from clinical records and routine radiological imaging examinations. Preoperative chemotherapy was defined as chemotherapy administered within 90 days before liver surgery. Postoperative chemotherapy was determined as chemotherapy administered within 90 days after liver surgery. Prolonged preoperative chemotherapy was specified as more than 6 cycles. Postoperative CT was used routinely in the follow-up program for detecting disease recurrence, and the same investigation was used for diagnosing IH. The location of IH was categorized as upper/lower midline and subcostal.

The most recent CT before liver surgery and the most recent CT at the time of data collection were analyzed by a single investigator. IH was defined as a discontinuity in the abdominal fascia observed on CT scan [2, 13]. The presence of IH after previous abdominal surgery on preoperative CT was recorded.

The abdominal wall-specific questionnaire used was the validated ventral hernia pain questionnaire (VHPQ) [12, 14]. It consists of 19 questions that reflect the patients’ perception of the incisional site concerning pain, cosmetic issues, social limitations and abdominal wall stiffness. Questions concerning the level, frequency and duration of pain, use of pain medication, and impact on daily activities were also included. Clinically significant symptoms were determined as: pain right now/last week-not easily ignored, scar cosmetically disturbing, scar socially limiting, and abdominal wall stiffness.

The EORTC QLQ-C30 is a 30-item questionnaire composed of multi-item scales and single items that reflect QoL. It includes 5 functional domains (physical, role, emotional, cognitive, and social functioning), a global health score, and 9 cancer-related symptoms [11]. For the five functional scales and global health, a high score represents a high level of functioning, whereas for the symptom scales, a high score indicates a high level of symptoms. Missing data were handled according to the instructions from the validated manual [11]. Clinical importance of differences in QoL scores was defined as none (≤ 5), little (5.0–10.0), moderate (10.0–20.0) or large (> 20.0) [15].

The liver was accessed with a right subcostal incision, 4–5 cm caudal of the costal margin with a midline cranial extension to the xiphoid process, usually measuring 4–8 cm, defining an extended right subcostal incision (J-shaped incision). If necessary, the incision was extended to the left, resulting in a bilateral subcostal incision with a cranial midline extension (Mercedes incision). Liver transection was performed using a standardized technique, as previously described in detail [16]. A major resection was defined as resection of ≥ 3 Couinaud’s segments. The abdominal wall fascia was closed in two layers with a running, slowly absorbable PDS suture (Johnson & Johnson, Diegem, Belgium) and the skin was stapled. No drains were used.

The study protocol was approved by the regional ethics committee. Dnr 2016/989.

### Statistical analysis

Data were expressed as numbers (percentages) or median (interquartile range). Results from the EORTC QLQ-C30 questionnaires were transformed into function and symptom scales (0-100) according to instructions from the validated manual [11]. The Mann-Whitney U test was used to compare continuous data, and Fisher’s exact test or the Chi square test was used for categorical data. Predictor variables with a *P* < 0.1 on univariate analysis were included in multivariate analysis. Multiple linear and logistic regression analyses were performed to identify parameters with a significant influence on abdominal wall symptoms and QoL. A *p* < 0.05 was considered statistically significant. Statistical analyses were performed using IBM SPSS statistics version 25 (IBM, Armonk, NY, USA).

## Results

A total of 131 patients who were operated on between January 2010 and December 2015 and were alive in February 2017 were included. Three patients actively refrained from participating in the study. One hundred and six patients returned completed questionnaires, one of which was excluded due to missing follow-up radiology. Thus, 105 patients were analyzed regarding IH, calculating a response rate of 80% (105/131). The median follow-up was 34 months.

In total, 43 patients (42%) developed IH during the study period. Patient characteristics and perioperative data for the IH and non-IH groups are shown in Table 1. Most hernias measured less than 2.5 cm, six measured 2.5–4.5 cm and four measured > 4.5 cm. The IH was located in the midline above the umbilicus in 30/43 patients. Six patients had IH involving a subcostal incision, five of which were located within the rectus sheath, and one lateral to the rectus sheath. Additionally, 7/43 patients had IH in the umbilicus and below the umbilicus related to previous laparotomies.

**Table 1:** Characteristics of patients with and without incisional hernia

Variable	Number of analyzed patients	Incisional hernia *N* = 43	No incisional hernia *N* = 62	*P*
Male gender	105	27 (63%)	35 (57%)	0.619
Age (years)	105	69 (56–73)	62 (55–74)	0.709
Current smoking	105	9 (21%)	15 (25%)	0.619
ASA grade ≥ 3	105	9 (21%)	16 (26%)	0.685
Diabetes mellitus	105	2 (5%)	9 (15%)	0.115
Body mass index (kg/m^2^)	105	25.0 (24.5–27.7)	25.2 (22.5–27.3)	0.056
Preoperative albumin (g/l)	105	36 (39–40)	35 (33–38)	0.706
Preoperative chemotherapy	105	21 (49%)	36 (59%)	0.252
Preoperative Bevacizumab	104	2 (5%)	1 (2%)	0.549
Number of chemotherapy cycles	103	5 (4–6)	5 (4–6)	0.439
Chemotherapy cycles > 6	103	4 (9%)	3 (9%)	0.408
Synchronous liver and primary tumors	97	23 (54%)	30 (56%)	1.000
Synchronous liver and lung tumors	92	6 (14%)	1 (2%)	0.039
Rectal primary	97	14 (14%)	18 (33%)	0.936
Major liver resection	105	17 (16%)	30 (49%)	0.284
Operating time (h)	105	3.5 (3.0–7.0)	6.0 (5.0–7.0)	0.386
Bleeding (ml)	104	500 (175–1100)	500 (300–700)	0.444
Incisional infection	104	4 (4%)	3 (5%)	0.444
Hospital-stay (days)	105	6 (5–8)	7 (6–9)	0.287
Clavien-Dindo ≥ 3	105	7 (16%)	4 (7%)	0.195
Postoperative chemotherapy	104	27 (63%)	52 (87%)	0.003
Alive with recurrent disease	105	10 (23%)	11 (18%)	0.553
Median follow up time (months)	105	33 (21–52)	24 (20–38)	0.216
Operation for incisional hernia	105	2 (5%)	1 (2%)	0.566
Incisional hernia before liver surgery	105	13 (30%)	3 (5%)	0.001
One or more incisions besides liver surgery	105	36 (86%)	54 (87%)	0.627
Two or more incisions besides liver surgery	105	9 (21%)	9 (15%)	0.391

Incisions for liver resection were an extended right subcostal J-shaped incision (72 patients, 69%), right subcostal only (13 patients, 12%), Mercedes incision (14 patients, 13%), bilateral subcostal (2 patients, 2%), and midline incision only (1 patient, 1%),

At the time of evaluation, 90 patients (86%) had other abdominal incisions in addition to the current liver surgery. Eighty-five patients (81%) had midline incisions, and three patients (3%) had undergone an open cholecystectomy.

At the end of follow-up, 18 patients (17%) had had two or more additional abdominal incisions, three patients (3%) had been re-operated for IH, and 9 patients (9%) had experienced an infection at the incision site. Ten patients (10%) had undergone re-resection of the liver.

Forty-nine patients (48%) reported at least one significant abdominal wall symptom. Twenty-one patients in the IH group and 28 patients (45%) in the non-IH group experienced clinically significant abdominal wall symptoms (*P* = 0.547). There was no difference in global QoL (*P* = 0.687) or significant abdominal wall symptoms (*P* = 0.721) in patients with IH of different sizes (that is < 2.5 cm, 2.5–4.5 cm and > 4.5 cm). The prevalence and distribution of the different symptoms reported in VHPQ are showed in Table 2. The distribution of QoL from EORTC QLQ-C30 is shown in Table 3.

**Table 2:** Distribution of abdominal wall symptoms in patients with and without incisional hernia

Clinically significant symptoms	Number of analyzed cases	Incisional hernia *N* = 41	No incisional hernia *N* = 62	*P*
Any significant symptom	103	21 (51%)	28 (45%)	0.547
Pain right now-not easily ignored	100	12 (29%)	13 (22%)	0.411
Pain last week-not easily ignored	100	11 (26%)	15 (25%)	0.875
Scar cosmetically disturbing	102	9 (22%)	7 (11%)	0.129
Scar socially limiting	102	4 (10%)	5 (9%)	0.737
Abdominal wall stiffness	101	12 (29%)	15 (25%)	0.602

**Table 3:** Distribution of EORTC QLQ C-30 variables between groups

Quality of life	Number of analyzed cases	Incisional hernia *N* = 43	No Incisional hernia *N* = 62	*P*
Global quality of life	104	75.0 (50.0-93.8)	83.3 (66.7–100.0)	0.131
Physical function	105	90.0 (60.0-100.00)	93.3 (73.3–100.0)	0.346
Role function	105	100 (66.7–100.0)	100 (66.7–100.0)	0.960
Cognitive function	105	83.3 (83.3–100.0)	83.3 (79.2–100.0)	0.466
Emotional function	105	91.7 (75.0-100.0)	91.7 (72.9–100.0)	0.902
Social function	105	91.7 (66.7–100.0)	100 (66.7–100.0)	0.355
Fatigue	104	33.3 (8.3–44.4)	33.3 (11.1–33.3)	0.790
Nausea and vomiting	104	0.0 (0.0–0.0)	0.0 (0.0–0.0)	0.559
Pain	104	0.0 (0.0-16.7)	0.0 (0.0-16.7)	0.704
Dyspnea	104	33.3 (0.0-33.3)	16.7 (0.0-33.3)	0.994
Sleep disturbance	104	16.7 (0.0-33.3)	0.0 (0.0-33.3)	0.381
Appetite loss	105	0.0 (0.0–0.0)	0.0 (0.0–0.0)	0.968
Constipation	104	0.0 (0.0-33.3)	0.0 (0.0-33.3)	0.740
Diarrhea	104	0.0 (0.0-33.3)	0.0 (0.0–0.0)	0.149
Financial impact	103	0.0 (0.0–0.0)	0.0 (0.0–0.0)	0.674

Pooling the results of the VHPQ, concerning the level and duration of pain as well as questions related to the impact on daily living, showed the following:

Ninety-one (87%) patients no longer experienced pain from the scar after liver surgery. Among these patients, pain had ceased within one month after surgery in 54/91 (59%), within 2–3 months in 24/91 (26%) and within 4–6 months in 7/91 (8%). Three patients experienced pain relief more than one year after surgery.

Patients who still experienced pain from the incision were asked to answer the remaining questions in the form, which addressed the nature of the pain. Ten patients responded to questions about the nature of the pain. Five reported excruciating pain once a week, one patient experienced pain 2–5 times a week, two patients had intermittent daily pain, one patient had pain every day, including at night, and one patient had continuous pain.

Multivariate analyses of the VHPQ showed that the most powerful determinant for pain symptoms was having undergone 2 or more laparotomies in addition to liver surgery (Odds ratio 4.17, 95% confidence interval 1.17–14.86, *p* = 0.028). However, no significant correlation between pain symptoms and IH was found (*p* = 0.411).

## Discussion

In the present single-center observational study, which reports on abdominal wall symptoms and QoL for patients resected for colorectal liver metastases, we did not find significant differences between the IH and non-IH groups. This finding is consistent with a previous study investigating abdominal wall symptoms in patients treated for open abdomen [14], which showed no differences in scores between patients with and without IH at a 5-year follow-up. However, our results contrast with some other published data [17, 18, 19], which showed deterioration of both QoL scales and body image. The differences might be explained by different detection methods for IH and patient selection.

The first study [17], one of the few existing reports analyzing the impact of IH on QoL in patients who had not yet been selected for hernia repair, revealed that the presence of IH significantly impacted QoL, including decreased role physical and physical component summary scores, as well as a decline in almost all scores concerning body image. All patients in that study underwent physical examination to detect IH, whereas in our study, all IHs were detected via CT. Radiological imaging has been shown to detect more hernias than clinical examination alone [5].

The second study [18], showed that all eight health-related domains for patients with IH were significantly lower than those in a healthy control group. However, this group of patients had already been selected for hernia repair, so it is conceivable that these patients had more pronounced symptoms from the outset. The latest study [19], showed that occurrence of IH was predictive of lower QoL scores in four of the eight domains, including physical function, general health, vitality, and social function. Additionally, the IH group displayed a lower mental health score and mental component summary score. In this study, surgery-related complications, such as IH, were detected clinically with questionnaires and subsequent telephone interviews. These findings suggest that IH detected solely by CT may have less clinical importance.

Notably, the current study did not show pain as the predominant symptom for patients with IH. Our data align with results from previous studies [14, 17, 19] which did not report any significant differences regarding pain for the different groups with and without IH.

In our study, the most significant determinant for pain symptoms was the number of laparotomies performed in addition to liver surgery. The impact of multiple surgical incisions is consistent with previous findings, where three or more abdominal operations predisposed patients to gastrointestinal complaints and abdominal pain six months after open surgery [20].

The present study demonstrated an IH incidence of 42%, despite abdominal wall closure for subcostal incision being performed in two layers and one layer in midline with a running, slowly absorbable monofilament suture, which has been suggested to prevent incision hernias [21]. The incidence is in line with a previous report using CT as detection method [1], although it is higher than reported in another study [22]. The difference is likely due to the use of CT as a detection method [5]. Previous surgical procedures in most patients may have been a confounding factor. Additionally, the longer study period, with median follow-up of 34 month, could contribute to the high IH incidence, as 75% of all IHs develop after 2 years and about 90% after 5 years [23]. Our study also showed that patients who had an IH before the current liver surgery developed a new IH more often (*P* = 0.001). This finding is consistent with previous observations [4].

The majority of IH were small (< 2.5 cm), only 4 hernias were measured as large as around 5 cm. Although common diagnostic criteria for IH are lacking, different classifications of IH have been proposed in the literature, often related to the size of the hernia as measured by the width of defect in the abdominal wall (W1 < 4 cm; W2 ≥ 4–10 cm; W3 ≥ 10 cm) [24].

This study showed that a vast majority, 70%, of IHs were located in the midline above the umbilicus, and about 14% were within the subcostal incision. These findings align with previous studies [4, 21], which demonstrate that a transverse incision is preferable to a midline incision in terms of IH development. Nevertheless, approximately half (48%) of all patients experienced some clinically significant abdominal wall symptoms after a median follow-up of 34 months, although this was not related to IH.

Furthermore, no difference in abdominal wall complaints was found between patients with and without IH. A systemic review [25] reported that approximately half of the patients with IH have symptoms. In this study, only 3 out of 105 patients were operated on for IH during the study period. This is substantially lower than previously reported [14, 25], and it has been suggested that surgical repair of IH in these patients would have a limited effect on their QoL. The aim of the present study was not to investigate the rate of IH repair, as this decision is based on clinical symptoms rather than radiological diagnosis, which would require longitudinal follow up.

A sub-analysis of the VHPQ showed that pain from the abdominal incision disappeared in most patients (59%) within one month after liver surgery and in another 26%, it disappeared within 2–3 months. Most of the remaining patients did not report pain after 6–12 months. These results are consistent with the validation study of the VHPQ, which reported that 22 out of 51 patients (43%) who underwent surgery for ventral hernia experienced pain relief after 4 weeks [12]. Although the VHPQ was developed and validated for assessing pain following surgery for ventral hernia, it was used in the present study for measuring pain after liver surgery, a procedure for which VHPQ has not been validated. This should be considered when comparing the results of the present study to other studies using the VHPQ.

Analysis of patient-reported outcomes after liver surgery for colorectal liver metastases has shown a decrease in most functional aspects of health and an increase in symptoms up to three months after surgery [26]. Functional scales improved within 6 months and were maintained at one year, while symptom scales, including pain, returned to baseline levels at 12 months. About 12% of patients experienced abdominal pain, which is similar to our study, where 10 of 105 patients reported persistent pain when filling out the form. The prevalence of chronic pain, as analyzed with QoL and specific pain questionnaires, has been reported as high as 18% four years after gastrointestinal surgery [27]. Similar numbers have been reported in studies analyzing QoL after emergency laparotomy [28, 29].

The main limitations of the present study are the lack of a control group and the limited number of included patients. Additionally, no measurement of QoL was performed before surgery. During the study period, patients underwent various laparotomies in addition to the present liver surgery, making it difficult to distinguish whether the abdominal symptoms and changes in QoL scales were related specifically to liver surgery. Furthermore, the response rate to the questionnaires (80%) may have affected the interpretation of the results.

The use of minimally invasive surgical techniques has been shown to reduce deterioration in QoL after liver surgery in the short term [30]. Patients in the laparoscopic group reported better scores up to four months after surgery compared to open surgery. However, the possible long-term impact on QoL remains to be shown and must be considered in conjunction with concurrent surgery.

## Data Availability

No datasets were generated or analysed during the current study.
